# Effectiveness and Safety of Endoscopic Submucosal Dissection for Colorectal Neoplasm in Patients with High Charlson Comorbidity Index Score: A HASID Multicenter Study

**DOI:** 10.3390/jcm12196255

**Published:** 2023-09-28

**Authors:** Dong-Hyun Kim, Yong-Wook Jung, Byung-Chul Jin, Hyung-Hoon Oh, Hyo-Yeop Song, Seong-Jung Kim, Dae-Seong Myung, Sang-Wook Kim, Jun Lee, Geom-Seog Seo, Young-Eun Joo, Hyun-Soo Kim

**Affiliations:** 1 Department of Internal Medicine, Medical School, Chonnam National University, Gwangju 61469, Republic of Korea; bono343@cnuh.com (D.-H.K.); gi00166@cnuh.com (Y.-W.J.); gi00163@cnuh.com (H.-H.O.); yejoo@chonnam.ac.kr (Y.-E.J.); 2 Department of Internal Medicine, Medical School, Jeonbuk National University, Jeonju 54907, Republic of Korea; 23743@jbuh.co.kr (B.-C.J.); clickm@jbnu.ac.kr (S.-W.K.); 3 Department of Internal Medicine, Digestive Diseases Research Institute, School of Medicine, Wonkwang University, Iksan 54538, Republic of Korea; bfsongc@hanmail.net (H.-Y.S.); medsgs@wonkwang.ac.kr (G.-S.S.); 4 Department of Internal Medicine, College of Medicine, Chosun University, Gwangju 61452, Republic of Korea; ygegh@chosun.ac.kr (S.-J.K.); leejun@med.chosun.ac.kr (J.L.)

**Keywords:** aged, colon, comorbidity, endoscopic submucosal dissection

## Abstract

Endoscopic submucosal dissection (ESD) is an effective method for removing early colorectal lesions. However, research on the safety and efficacy of ESD in patients with various underlying conditions remains limited. This study retrospectively examined ESD outcomes in colorectal neoplasm patients from five tertiary medical centers. The Charlson Comorbidity Index (CCI) and age-adjusted CCI (ACCI) were analyzed, and the differences in complete resection and complication rates were analyzed. The CCI, ACCI, and complication rates tended to gradually increase proportionally, and the complication resection rate increased from CCI 2 to ACCI 4 as the starting point, followed by a decreasing trend. Of these, 140 patients (9.7%) had a CCI score of 3 or higher. The high CCI group was older (70.6% vs. 64.7%, *p* < 0.01) and had a higher proportion of men (70.7% vs. 58.7%, *p* < 0.01) than the low CCI group. The high CCI group had a higher incidence of cancer than the low CCI group (77.9% vs. 65.2%, *p* < 0.01). The en bloc resection rate (90.0% vs. 89.3%, *p* = 0.79) and complete resection rate (75.7% vs. 81.2%, *p* = 0.12) were not significantly different between the two groups. Colorectal ESD can be safely and effectively performed in patients with various underlying medical conditions.

## 1. Introduction

The detection rate of colorectal cancer is increasing with the development of colonoscopy, early screening, and the discovery of early lesions in colon neoplasms [1,2]. Endoscopic submucosal dissection (ESD), which enables en-bloc resection of large tumors, is the standard minimally invasive treatment for early colorectal cancer. Intramucosal tumors with non-lifting signs and 0-IIc lesions >20 mm are particularly well suited for colorectal ESD, as this technique allows for meticulous dissection and en-bloc removal of such challenging lesions, thereby improving the chances of complete tumor eradication and minimizing the risk of recurrence. Additionally, sizable sessile lesions that pose challenges for en-bloc resection through conventional endoscopic mucosal resection (EMR) can be considered suitable candidates for colorectal ESD [3,4].

According to the Korean Clinical Practice Guidelines for Endoscopic Resection of Early Gastrointestinal Cancer, ESD is preferred over EMR for early colorectal cancer [5]. The European Society of Gastrointestinal Endoscopy recommends a laterally spreading tumor lesion size > 20 mm for a patient to be eligible for removal using ESD [6].

In real-world clinical settings, endoscopists must consider comorbidities, performance status, and expected survival [7,8]. Researchers widely utilize the Charlson Comorbidity Index (CCI) to assess the complex burden of comorbidities [9]. CCI helps predict mortality by categorizing or assigning weights to different comorbidities. This index has been validated across a range of disease subgroups, including cardiac disease [10], liver disease [11], malignancy [12], and COVID-19 [13].

The age-adjusted CCI (ACCI) is a value that increases by 1 every 10 years when the patient is 50 years or older and is calculated by adding it to the original CCI. This number is also determined by calculating the factor of age together with the underlying disease [14]. Recent studies have reported that ACCI can be used to predict the prognosis of many diseases [15,16,17,18]. However, despite these findings, the relationship among comorbidities, treatment outcomes, and complications of endoscopic resection is not well established. The present study aimed to elucidate the relationship between comorbidities, treatment outcomes, and complications associated with ESD for colorectal neoplasms.

## 2. Materials and Methods

### 2.1. Study Design, Setting, and Patients

This multicenter, retrospective study used data collected between January 2015 and December 2020. Data were collected from five tertiary medical centers (Chonnam National University Hospital, Chonnam National University Hwasun Hospital, Jeonbuk National University Hospital, Chosun University Hospital, and Wonkwang University Hospital) in the Honam Province, South Korea. A total of 1446 patients who underwent endoscopic submucosal resection of colorectal epithelial tumors were analyzed. We reviewed the medical records and extracted information on the demographic and clinical characteristics, procedural outcomes, and procedure- or sedation-related complications of colorectal ESD.

This retrospective multicenter study obtained the necessary ethical approval from the respective institutional review boards and ethics committees in accordance with the principles outlined in the Declaration of Helsinki. The study protocol and data collection procedures were reviewed and deemed ethically acceptable, taking into consideration the rights, welfare, and privacy of the study participants. All patient information used in this study was de-identified and handled with strict confidentiality. The study was conducted in compliance with the applicable regulations, guidelines, and ethical standards to ensure the integrity and validity of the research findings.

### 2.2. Data Collection and Outcomes

Data on age, sex, location, gross morphology, lesion size, procedure time, pathological findings, presence of fibrosis, adverse events, and complete resection rates were collected. The lesion location was categorized into three groups: right colon (cecum, ascending colon, and transverse colon), left colon (descending colon and sigmoid colon), and rectum. The macroscopic appearance of lesions was classified as protruded, flat-elevated, or flattened/depressed according to the Paris classification [19]. En-bloc resection was performed when the lesion was resected in a single piece. Complete resection was defined as the histological base, and the lateral margin was negatively resected in a single piece. Anticoagulant and antiplatelet therapies were discontinued for the duration advised before ESD [20]. The assessment of ESD as a procedure with a high bleeding risk prompted the discontinuation of the medication. Discontinuation decisions were personalized based on the patient’s existing thromboembolic status.

Patients were sedated with midazolam, propofol, or both. Supplementary oxygen was administered to maintain arterial oxygen saturation > 90%. Blood pressure, heart rate, and oxygen saturation levels were monitored during the procedure. Hypotension during the procedure was characterized by a systolic blood pressure below 90 mmHg. Oxygen desaturation was defined as an arterial oxygen saturation of less than 90%, persisting for a minimum of 10 s. An antidote for midazolam (flumazenil^®^) was administered when a paradoxical reaction or desaturation was observed during sedative endoscopy. Procedure-associated bleeding was defined as bleeding necessitating transfusion, urgent endoscopy, or a decrease in hemoglobin levels by more than 2 g/dL after the procedure [21]. Procedure-related perforation was defined as an endoscopically observed extraluminal space or intra-abdominal free air on radiography or computed tomography performed after the procedure [22]. Post-ESD coagulation syndrome (PECS) after colonic ESD is described as the manifestation of inflammatory markers, including procedure-associated abdominal tenderness, fever (≥37.6 °C), leukocytosis (≥10,000 cells/μL), or elevated C-reactive protein levels (≥0.5 mg/dL). This definition was applied to patients without indications of post-ESD perforation [23].

We examined the presence or absence of each underlying disease and computed the CCI scores. The presence or absence of each underlying medical condition was examined, and CCI scores were computed. The components of CCI included a history of myocardial infarction, congestive heart failure, peripheral vascular disease, cerebrovascular accident, dementia, chronic obstructive pulmonary disease, connective tissue disease, peptic ulcer disease, diabetes, renal disease, hemiplegia, leukemia, lymphoma, solid tumors with or without metastatic presence, liver disease, and acquired immunodeficiency syndrome. CCI was determined by summing the weights assigned to all comorbid parameters. The total CCI score was 0–33 [9]. The ACCI is a score in which the value increases by 1 point for each increase in age by 10 years from less than 50 years of age, with 0 points for the above score [14]. Clinical characteristics and ESD results were compared between the high CCI group (CCI ≥ 3) and the low CCI group (CCI < 3).

### 2.3. Statistical Analysis

Continuous data were presented as mean ± standard deviation or median (range), while categorical data were displayed as absolute or relative frequencies. The Student’s *t*-test was used for the analysis of continuous variables. Fisher’s exact test or the χ^2^ test was used for categorical data. Furthermore, Kaplan–Meier analysis was conducted to examine long-term follow-up data, encompassing recurrence and mortality. Statistical significance was defined as a *p*-value less than 0.05. All statistical analyses were conducted using IBM SPSS version 25 (IBM Corp., Armonk, NY, USA).

Experiments were conducted using restricted cubic splines with three knots. A restricted cubic spline curve was used to show the predicted probability (solid line) and 95% confidence intervals (shades) to evaluate the association of CCI and ACCI with complete resection and complication rates. Statistical analyses were conducted using Stata/SE 16.1 software (StataCorp LLC., College Station, TX, USA).

## 3. Results

### 3.1. Patients and Comorbidities

**Figure 1:**
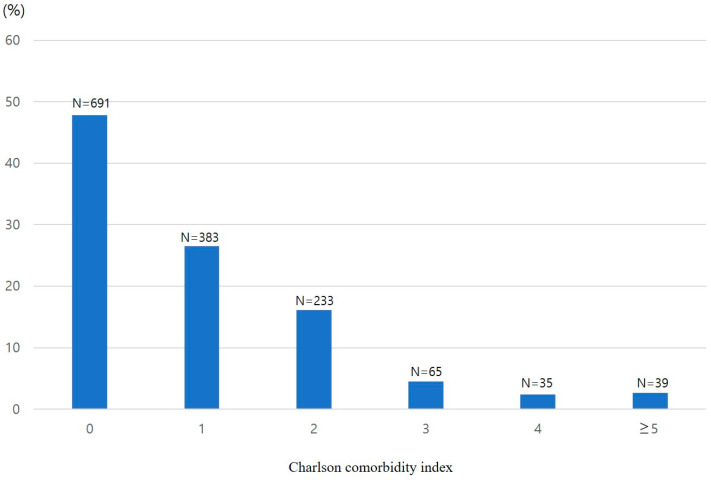
Percentage distribution of Charlson Comorbidity Index scores in patients undergoing colorectal endoscopic submucosal dissection.

**Figure 2:**
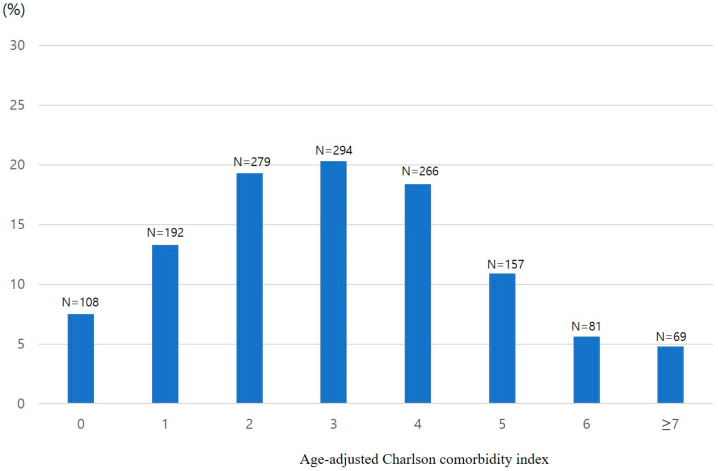
Percentage distribution of age-adjusted Charlson Comorbidity Index scores among patients undergoing colorectal endoscopic submucosal dissection.

There were a total of 1446 patients. The most common comorbidity was diabetes (21.2%), followed by solid tumors without recurrence (9.7%). Table 1 lists the comorbidities of the patients. According to the CCI, the distribution of the corresponding number of patients and percentages were as follows: 0 points, *n* = 691, 47.8%; 1 point, *n* = 383, 26.5%; 2 points, *n* = 233, 16.1%; 3 points, *n* = 65, 4.5%; 4 points, *n* = 35, 2.4%; and ≥5 points, *n* = 39, 2.7% (Figure 1). Similarly, based on the ACCI, the corresponding number of patients and percentages were as follows: 0 points, *n* = 108, 7.5%; 1 point, *n* = 192, 13.3%; 2 points, *n* = 279, 19.3%; 3 points, *n* = 294, 20.3%; 4 points, *n* = 266, 18.4%; 5 points, *n* = 157, 10.9%; 6 points, *n* = 81, 5.6%; and ≥7 points, *n* = 69, 4.8% (Figure 2).

### 3.2. Procedures

A total of 1446 patients with colorectal neoplasm (mean age of 65.3 ± 11.2 years; 581 females, 40.2%) were included in the final analysis. The mean longitudinal tumor size was 29.6 ± 12.3 mm). Colorectal neoplasms were located in the cecum and were ascending in 486 (33.6%) patients, transverse and descending in 322 (22.3%) patients, and sigmoid and rectal in 638 (44.1%) patients. The mean procedure time was 47.4 ± 46.4 min. Histological examination revealed 774 adenomas (53.5%), 300 intramucosal cancers (20.7%), and 372 invasive cancers (25.7%). The intake rates of aspirin, clopidogrel, and antithrombotic medications were 8.8%, 3.5%, and 1.0%, respectively. Sedative endoscopy was performed in 70.4% of the patients. Midazolam was administered to only 942 patients (65.1%), followed by midazolam and propofol (71 patients, 4.9%) and propofol monotherapy (5 patients, 0.3%).

Table 2 summarizes the differences in demographics, size, location, histology, medication, and sedation between the two groups (high and low CCI groups). There was a difference in age between patients with CCI ≥ 3 (70.6 ± 8.8 years) and patients with CCI < 3 (64.7 ± 11.2 years, *p* < 0.01). Males were more dominant in the high CCI group (70.7%) than in the low CCI group (58.7%, *p* < 0.01). Advanced histologic lesions (intramucosal or invasive cancer) were more frequent in the high CCI group (77.9%) than in the low CCI group (65.2%; *p* < 0.01). Aspirin (25.0% vs. 7.0%, *p* < 0.01) and clopidogrel (10.7% vs. 2.7%, *p* < 0.01) were more frequently described in the high CCI group than in the low CCI group. Sedative endoscopy was performed less frequently in the high CCI group (57.9%) than in the low CCI group (71.7%; *p* < 0.01).

### 3.3. Treatment Outcome and Complications

**Figure 3:**
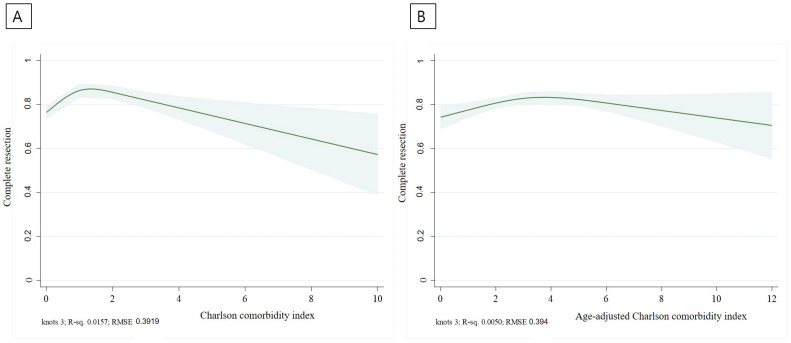
Complete resection prediction model according to CCI (**A**) and ACCI (**B**).

**Figure 4:**
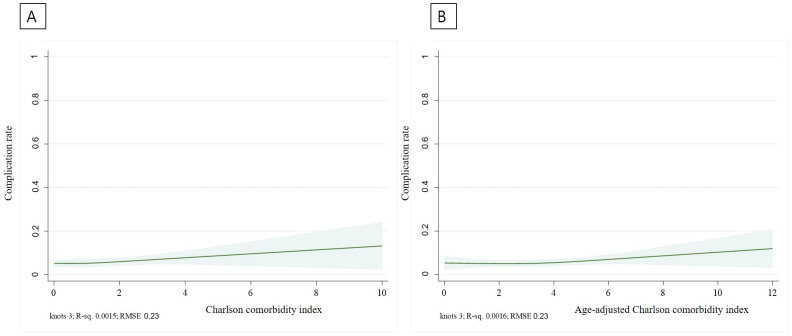
Complication occurrence prediction model according to CCI (**A**) and ACCI (**B**).

We plotted a restricted cubic spline curve representing the predicted probability (solid line) and 95% confidence intervals (shades). Figure 3 shows the complete resection rate prediction model based on the CCI and ACCI. The complete resection rate tended to have the highest CCI score of 1 and gradually decreased toward a high CCI score. When analyzed with ACCI, the complete resection rate was the highest in ACCI 3–4, which showed a gradual decrease. As shown in Figure 4, bleeding, perforation, and PECS were regarded as complications, and the complication rate gradually increased according to CCI and ACCI values. Table 2 summarizes the treatment outcomes and complications observed in the two groups. En-bloc and complete resections were achieved in 89.3% and 80.7% of the patients, respectively. En-bloc resection was performed in 90.0% and 89.3% of the high and low CCI group patients, respectively (*p* = 0.79). Complete resection was performed in 75.7% and 81.2% of patients in the high and low CCI groups, respectively (*p* = 0.12).

Perforation occurred in 0.6% of participants. The perforation rate (0.7% vs. 0.6%, *p* = 0.88) did not differ significantly between the high and low CCI groups. Bleeding occurred in 2.4% of patients. The incidence of bleeding (3.6% vs. 2.2%, *p* = 0.32) did not differ significantly between the high and low CCI groups. PECS occurred in 2.8% of participants. PECS was similar in both groups (2.9% vs. 2.8%, *p* = 0.99). Hypotension (0% vs. 0.1%, *p* = 0.74) and desaturation (0% vs. 0%) during sedative endoscopy have rarely been reported.

### 3.4. Long-Term Follow-Up

Follow-up endoscopy was conducted in 978 out of 1446 patients. The mean duration of endoscopic follow-up was 24 months, with a median duration of 16 months (range, 1–94 months). On average, patients underwent follow-up endoscopy 2.1 times, with a median of two times and a range spanning from one to 11 times. Among these, recurrence of colorectal neoplasms was observed in 20 patients (2.0%). Among patients confirmed with recurrence, surgery was performed in 12 (60%), additional ESD in 5, and the remaining 3 were followed up without further surgical intervention. Among the 978 patients who underwent follow-up endoscopy, 94 exhibited a CCI score of 3 or higher, while 884 had a CCI score below 3. In the high CCI group, encompassing 94 patients, the average duration of endoscopic follow-up amounted to 27.8 months, with a median duration of 23 months and a range spanning from 1–79 months. The number of follow-up endoscopies varied between one and seven, averaging 2.5 times and having a median of two times. Among the 94 patients in the high CCI group, colorectal neoplasm recurrence was verified in three (3.2%) patients. Within the low CCI group encompassing a total of 884 patients, the mean duration of endoscopy follow-up was 23.6 months, with a median duration of 14 months and a range spanning from 2–94 months. The frequency of follow-up endoscopies ranged from 1–11, with a median of 1 endoscopy and an average of 2. Colorectal neoplasm recurrence was detected in 17 of the 884 patients, constituting a rate of 1.9%. Assessing recurrence through Kaplan–Meier analysis with a log-rank test between the two groups revealed a *p*-value of 0.63, indicating no significant disparity in recurrence based on CCI.

Among the 1446 patients, outpatient follow-up was conducted in 1412 patients. The average follow-up period was 21.4 months (median, 14 months; range, 1–107 months). During the follow-up period, five patients experienced mortality, accounting for 0.4% of the cohort. The causes of death included lung cancer, worsening of idiopathic pulmonary fibrosis, heart failure, aplastic anemia, and acute cholangitis, each occurring in one case. Importantly, no deaths attributed to colorectal neoplasms were recorded. In the high CCI group, comprising 135 patients, four died during the follow-up period, accounting for a mortality rate of 3.0%. The causes of death included lung cancer, heart failure, aplastic anemia, and acute cholangitis. In the low CCI group, which comprised 1277 patients, one died during the follow-up period. The cause of death was the exacerbation of idiopathic pulmonary fibrosis. Kaplan–Meier analysis with a log-rank test was performed to compare mortality rates between the high and low CCI groups, and the high CCI group demonstrated a significantly higher mortality rate (*p* < 0.01).

## 4. Discussion

Colorectal ESD is an important treatment option for early malignant neoplasms [21]. According to both the European Society of Gastrointestinal Endoscopy and Korean guidelines, ESD is an effective treatment method with the highest complete resection rate [5,6]. However, studies examining the effectiveness of ESD in patients with various underlying diseases are lacking. In 1987, Charlson conducted a study on one-year mortality using the CCI scoring system, specifically focusing on underlying diseases [9]. This index has since been used as a prognostic predictor for various diseases [10,13]. ACCI, with age as an added factor, is also used commonly as a prognostic predictor [14,24].

Over the years, there have been studies on the outcome of ESD according to CCI in the esophagus and stomach [25,26]. However, to the best of our knowledge, no large-scale study has been conducted to examine whether ESD of colorectal neoplasms can be performed safely and effectively according to the CCI and ACCI. Although both groups commonly undergo ESD, colonoscopy requires bowel preparation and is performed on relatively thin walls. Hence, the risk associated with colorectal ESD necessitates distinct confirmation, apart from gastric and esophageal ESD. Therefore, we analyzed the efficacy and safety of colorectal ESD procedures based on both CCI and ACCI.

We created a predictive model called restricted cubic splines to determine the incidence of complete resection and complications in patients with high CCI and ACCI scores. Restricted cubic splines serve as a valuable method for schematically analyzing complications and complete resections within the framework of our predictive model [27]. Restricted cubic splines enable substantial flexibility in illustrating the connection between the predictors and outcomes. In this study, we could discern the relationship between CCI and ACCI in relation to complete resection and complication rates.

In the case of CCI, a high complete resection rate was observed at a CCI score of 1, after which the complete resection rate decreased gradually. In the case of ACCI, the complete resection rate was higher with ACCI scores of 3–4, and the complete resection rate decreased gradually. The restriction cubic spline curve is a linear prediction model based on actual data. This is a predictive model based on real-world data. In our study, CCI of 1 and ACCI between 3 and 4 showed the highest complete resection rate.

An actual CCI score of 1 indicates a state with few underlying diseases. Therefore, it can be inferred that significant changes in the outcomes of colorectal ESD may not manifest until a low CCI score of 1 is achieved. The average age of 65 years in this study implies that the ACCI would likely be 1–2 points higher than the CCI. Consequently, this suggests that an ACCI score of 3–4 might not be sufficiently elevated to significantly impact the complete resection rate compared to an ACCI of 0–2. When the complete resection rate was investigated by dividing the CCI group into high (≥3) and low (<3) groups, no significant difference was observed between the two groups for each complication (75.7% vs. 81.2%, *p* = 0.12). The complication occurrence model helps predict the occurrence of perforation, bleeding, or PECS, which shows a gentle upward trend in both the CCI and ACCI as the score increases. Nonetheless, upon dividing the CCI groups into high (≥3) and low (<3), the examination of complication rates failed to reveal any statistically significant differences between the two groups for each complication (perforation 0.7% vs. 0.6%, *p* = 0.88; bleeding 3.6% vs. 2.2%, *p* = 0.32; PECS 2.9% vs. 2.8%, *p* = 0.99).

The high CCI group exhibited a greater proportion of patients taking antiplatelet agents, including aspirin and clopidogrel, than the low CCI group. This observation may be attributed to the fact that patients with multiple underlying conditions were taking various medications. Nevertheless, discontinuing aspirin one week before ESD did not result in a statistically significant increase in the post-ESD bleeding rate [28,29]. We discontinued existing antiplatelet therapy according to the cessation strategy [20]. Dividing the CCI score by three or fewer points revealed no statistically significant difference in the occurrence of each complication between the two groups, even though the high CCI group was prescribed more antiplatelet agents (Table 3).

The high CCI group underwent sedative endoscopy less frequently than the low CCI group (57.9% vs. 71.7%, *p* < 0.01). In patients with many underlying diseases, sedative endoscopy was performed in a relatively small number of patients owing to concerns about complications related to sedative endoscopy. In this study, complications related to sedative endoscopy were extremely low in both (high and low CCI groups). The comprehensive complication analysis did not include sedation-related complications such as desaturation because many patients did not undergo sedative endoscopy. Therefore, we did not include the contents of the overall complications section.

In this study, we collected data from a large number of patients from multiple centers. Historically, there had been no definitive findings regarding the underlying disease score and colorectal ESD treatment outcomes (complete resection rate and complications). When analyzed by dividing the high CCI group from the low CCI group, we found no difference in the complication rate higher than expected or in the complete resection rate.

The long-term outcome analysis indicated that the recurrence rate was not linked to a high CCI as per the Kaplan–Meier analysis (3.2% vs. 1.9%, *p* = 0.63). Among patients who experienced a recurrence, various treatment approaches were employed. However, the two groups had a notable disparity in all-cause mortality. Kaplan–Meier analysis revealed that the high CCI group exhibited a significantly higher mortality rate with statistical significance (3.0% vs. 0.1%, *p* < 0.01). Similar to this study, in cases where ESD of the esophagus and stomach was performed, high CCI was associated with decreased overall survival in long-term follow-up [25,26].

Initially, both the CCI and ACCI were devised as indices linked to the longitudinal survival rate based on the underlying disease scores [9,14]. However, the CCI score also reflects prognoses for diverse conditions, finding utility as an indicator that encompasses various prognostic implications, extending beyond long-term follow-up outcomes. In the context of gastric ESD, a study revealed that among patients aged over 75 years, the high CCI group had a higher incidence of respiratory complications, including atelectasis, than that in the low CCI group [30]. In cases of colonic diverticular bleeding, higher CCI scores are associated with an increased likelihood of severe diverticular bleeding, requiring interventions such as angioembolization or surgery [31]. Furthermore, studies have indicated that underlying conditions, such as cirrhosis, could elevate the complication rate of colonoscopic resections [32]. Reports have also indicated an elevated risk of bleeding during ESD procedures in patients with renal failure [33]. Consequently, exploring the correlation between a broad spectrum of underlying diseases and rates of complete resection and complications is necessary.

This study has a few limitations. This was a retrospective study. Retrospective studies often rely on existing data, which may introduce selection bias owing to the non-randomized nature of data collection. As the results of ESD conducted in five institutions in one country were shown, there was a limit to representing the situation by country or institution. Nevertheless, it has the advantage of showing the relationship between the underlying disease and ESD through a large number of multicenter studies.

## 5. Conclusions

The complete resection rate gradually decreased as the CCI and ACCI increased, and the incidence of complications gradually increased. When divided into the high CCI group and low CCI group, results showed that patients with a higher CCI score were older, had a higher incidence of cancer, and had higher intake rates of aspirin and clopidogrel. However, this study found no significant differences in the safety and efficacy of ESD between the high CCI group and low CCI groups. We conclude that colorectal ESD is a relatively safe and effective procedure despite various underlying diseases.

## Figures and Tables

**Table 1:** Baseline characteristics and comorbidities of a total of 1446 patients who underwent endoscopic submucosal dissection for colorectal neoplasm.

Variable		
Comorbidities, variable of CCI, *n* (%)	Weighting	
Acute myocardial infarction	1	65 (4.5)
Congestive heart failure	1	22 (1.5)
Peripheral vascular disease	1	7 (0.5)
Cerebrovascular disease	1	44 (3.0)
Dementia	1	16 (1.1)
Connective tissue disease	1	5 (0.3)
Chronic pulmonary disease	1	15 (1.0)
Peptic ulcer	1	2 (0.1)
Mild liver disease	1	40 (2.8)
Moderate to severe liver disease	3	11 (0.8)
Diabetes without end organ damage	1	284 (19.6)
Diabetes with end organ damage	2	22 (1.5)
Hemiplegia	2	2 (0.1)
Moderate to severe renal disease	2	25 (1.7)
Any solid tumor without metastasis	2	140 (9.7)
Leukemia	2	1 (0.1)
Lymphoma	2	3 (0.2)
Metastatic solid tumor	6	4 (0.3)
Acquired immunodeficiency syndrome	6	0
CCI, mean ± SD		0.98 ± 1.34
Age-adjusted CCI, mean ± SD		3.13 ± 1.96

SD, standard deviation; CCI, Charlson Comorbidity Index.

**Table 2:** Baseline characteristics for colorectal endoscopic submucosal dissection in the high Charlson Comorbidity Index group (CCI ≥ 3) and low Charlson Comorbidity Index group (CCI < 3).

	All Patients (*n* = 1446)	CCI ≥ 3 (*n* = 140)	CCI < 3 (*n* = 1306)	*p*-Value
Age, years	65.3 ± 11.2	70.6 ± 8.8	64.7 ± 11.2	<0.01
Female	581 (40.2)	41 (29.3)	540 (41.3)	<0.01
Tumor size, mm				
Long axis (mm)	29.6 ± 12.3	29.3 ± 13.8	29.6 ± 12.1	0.77
Short axis (mm)	25.1 ± 10.3	25.3 ± 10.5	25.1 ± 10.3	0.77
Tumor location				<0.01
Right side	486 (33.6)	31 (22.1)	455 (34.8)	
Left side	322 (22.3)	48 (34.3)	274 (21.0)	
Rectum	638 (44.1)	61 (43.6)	577 (44.2)	
Procedure time (min)	47.4 ± 46.4	42.2 ± 36.6	47.9 ± 27.3	0.09
Histology, *n* (%)				<0.01
Adenoma	774 (53.5)	31 (22.1)	455 (34.8)	
Intramucosal cancer	300 (20.7)	48 (34.3)	274 (21.0)	
Invasive cancer	372 (25.7)	61 (43.6)	577 (44.2)	
Medication, *n* (%)				
Aspirin	127 (8.8)	35 (25.0)	92 (7.0)	<0.01
Clopidogrel	50 (3.5)	15 (10.7)	35 (2.7)	<0.01
Antithrombotics	14 (1.0)	3 (2.1)	11 (0.8)	0.14
Sedation, *n* (%)	1018 (70.4)	81 (57.9)	937 (71.7)	<0.01
Midazolam	942 (65.1)	78 (55.7)	864 (66.2)	
Propofol	5 (0.3)	0	5 (0.4)	
Midazolam and Propofol	71 (4.9)	3 (2.1)	68 (5.2)	

Data are shown as mean ± standard deviation or *n* (%); CCI, Charlson Comorbidity Index.

**Table 3:** Treatment outcome and complications according to CCI score group.

	Total (*n* = 1446)	CCI ≥ 3 (*n* = 140)	CCI < 3 (*n* = 1306)	*p*-Value
En bloc resection	1292 (89.3)	126 (90.0)	1166 (89.3)	0.79
Complete resection	1167 (80.7)	106 (75.7)	1061 (81.2)	0.12
Procedural complication				
perforation	9 (0.6)	1 (0.7)	8 (0.6)	0.88
bleeding	34 (2.4)	5 (3.6)	29 (2.2)	0.32
PECS	41 (2.8)	4 (2.9)	37 (2.8)	0.99
Sedative complications				
Hypotension	1 (0.1)	0	1 (0.1)	0.74
Desaturation	0	0	0	
Use of antidote	1 (0.1)	0	1 (0.1)	0.74
Death	0	0	0	0

Data are shown as mean ± standard deviation or *n* (%); CCI, Charlson comorbidity index; PECS, post-endoscopic submucosal dissection coagulation syndrome.

## Data Availability

The data are not publicly available due to privacy and ethical restrictions. Data presented in this study are available upon request from the corresponding author.

## References

[1] Jemal A., Bray F., Center M.M., Ferlay J., Ward E., Forman D. (2011). Global cancer statistics. CA Cancer J. Clin..

[2] Jullumstrø E., Wibe A., Lydersen S., Edna T.H. (2011). Colon cancer incidence, presentation, treatment and outcomes over 25 years. Color. Dis..

[3] Saito Y., Otake Y., Sakamoto T., Nakajima T., Yamada M., Haruyama S., So E., Abe S., Matsuda T. (2013). Indications for and technical aspects of colorectal endoscopic submucosal dissection. Gut Liver.

[4] Hong S.W., Byeon J.S. (2022). Endoscopic diagnosis and treatment of early colorectal cancer. Intest. Res..

[5] Park C.H., Yang D.H., Kim J.W., Kim J.H., Kim J.H., Min Y.W., Lee S.H., Bae J.H., Chung H., Choi K.D. (2021). Clinical practice guideline for endoscopic resection of early gastrointestinal cancer. Intest. Res..

[6] Ferlitsch M., Moss A., Hassan C., Bhandari P., Dumonceau J.M., Paspatis G., Jover R., Langner C., Bronzwaer M., Nalankilli K. (2017). Colorectal polypectomy and endoscopic mucosal resection (EMR): European Society of Gastrointestinal Endoscopy (ESGE) Clinical Guideline. Endoscopy.

[7] Yoshida N., Naito Y., Sakai K., Sumida Y., Kanemasa K., Inoue K., Morimoto Y., Konishi H., Wakabayashi N., Kokura S. (2010). Outcome of endoscopic submucosal dissection for colorectal tumors in elderly people. Int. J. Color. Dis..

[8] Takahashi Y., Mizuno K.I., Takahashi K., Sato H., Hashimoto S., Takeuchi M., Kobayashi M., Yokoyama J., Sato Y., Terai S. (2017). Long-term outcomes of colorectal endoscopic submucosal dissection in elderly patients. Int. J. Color. Dis..

[9] Charlson M.E., Pompei P., Ales K.L., MacKenzie C.R. (1987). A new method of classifying prognostic comorbidity in longitudinal studies: Development and validation. J. Chronic Dis..

[10] Wellejus Albertsen L., Heide-Jørgensen U., Schmidt S.A.J., Grey C., Jackson R., Sørensen H.T., Schmidt M. (2020). The DANish Comorbidity Index for Acute Myocardial Infarction (DANCAMI): Development, Validation and Comparison with Existing Comorbidity Indices. Clin. Epidemiol..

[11] Jepsen P., Vilstrup H., Andersen P.K., Lash T.L., Sørensen H.T. (2008). Comorbidity and survival of Danish cirrhosis patients: A nationwide population-based cohort study. Hepatology.

[12] Kastner C., Armitage J., Kimble A., Rawal J., Carter P.G., Venn S. (2006). The Charlson comorbidity score: A superior comorbidity assessment tool for the prostate cancer multidisciplinary meeting. Prostate Cancer Prostatic Dis..

[13] Tuty Kuswardhani R.A., Henrina J., Pranata R., Anthonius Lim M., Lawrensia S., Suastika K. (2020). Charlson comorbidity index and a composite of poor outcomes in COVID-19 patients: A systematic review and meta-analysis. Diabetes Metab. Syndr..

[14] Charlson M., Szatrowski T.P., Peterson J., Gold J. (1994). Validation of a combined comorbidity index. J. Clin. Epidemiol..

[15] Koseki Y., Hikage M., Fujiya K., Kamiya S., Tanizawa Y., Bando E., Terashima M. (2021). Utility of a modified age-adjusted Charlson Comorbidity Index in predicting cause-specific survival among patients with gastric cancer. Eur. J. Surg. Oncol..

[16] Tian Y., Jian Z., Xu B., Liu H. (2017). Age-adjusted Charlson comorbidity index score as predictor of survival of patients with digestive system cancer who have undergone surgical resection. Oncotarget.

[17] Marventano S., Grosso G., Mistretta A., Bogusz-Czerniewicz M., Ferranti R., Nolfo F., Giorgianni G., Rametta S., Drago F., Basile F. (2014). Evaluation of four comorbidity indices and Charlson comorbidity index adjustment for colorectal cancer patients. Int. J. Color. Dis..

[18] Zhang N., Lin Q., Jiang H., Zhu H. (2023). Age-adjusted Charlson Comorbidity Index as effective predictor for in-hospital mortality of patients with cardiac arrest: A retrospective study. BMC Emerg. Med..

[19] Endoscopic Classification Review Group (2005). Update on the paris classification of superficial neoplastic lesions in the digestive tract. Endoscopy.

[20] Eisen G.M., Baron T.H., Dominitz J.A., Faigel D.O., Goldstein J.L., Johanson J.F., Mallery J.S., Raddawi H.M., Vargo J.J., Waring J.P. (2002). Guideline on the management of anticoagulation and antiplatelet therapy for endoscopic procedures. Gastrointest. Endosc..

[21] Tanaka S., Kashida H., Saito Y., Yahagi N., Yamano H., Saito S., Hisabe T., Yao T., Watanabe M., Yoshida M. (2020). Japan Gastroenterological Endoscopy Society guidelines for colorectal endoscopic submucosal dissection/endoscopic mucosal resection. Dig. Endosc..

[22] Kim S.J., Kim S.Y., Lee J. (2022). Prognosis and risk factors of electrocoagulation syndrome after endoscopic submucosal dissection in the colon and rectum. Large cohort study. Surg. Endosc..

[23] Yamasaki Y., Takeuchi Y., Iwatsubo T., Kato M., Hamada K., Tonai Y., Matsuura N., Kanesaka T., Yamashina T., Arao M. (2018). Line-assisted complete closure for a large mucosal defect after colorectal endoscopic submucosal dissection decreased post-electrocoagulation syndrome. Dig. Endosc..

[24] St-Louis E., Iqbal S., Feldman L.S., Sudarshan M., Deckelbaum D.L., Razek T.S., Khwaja K. (2015). Using the age-adjusted Charlson comorbidity index to predict outcomes in emergency general surgery. J. Trauma Acute Care Surg..

[25] Iwai N., Dohi O., Naito Y., Inada Y., Fukui A., Takayama S., Ogita K., Terasaki K., Nakano T., Ueda T. (2018). Impact of the Charlson comorbidity index and prognostic nutritional index on prognosis in patients with early gastric cancer after endoscopic submucosal dissection. Dig. Endosc..

[26] Nakajo K., Abe S., Oda I., Ishihara R., Tanaka M., Yoshio T., Katada C., Yano T. (2019). Impact of the Charlson Comorbidity Index on the treatment strategy and survival in elderly patients after non-curative endoscopic submucosal dissection for esophageal squamous cell carcinoma: A multicenter retrospective study. J. Gastroenterol..

[27] Croxford R. (2016). Restricted Cubic Spline Regression: A Brief Introduction.

[28] Lim J.H., Kim S.G., Kim J.W., Choi Y.J., Kwon J., Kim J.Y., Lee Y.B., Choi J., Im J.P., Kim J.S. (2012). Do antiplatelets increase the risk of bleeding after endoscopic submucosal dissection of gastric neoplasms?. Gastrointest. Endosc..

[29] Ono S., Fujishiro M., Niimi K., Goto O., Kodashima S., Yamamichi N., Omata M. (2009). Technical feasibility of endoscopic submucosal dissection for early gastric cancer in patients taking anti-coagulants or antiplatelet agents. Dig. Liver Dis..

[30] Kim S., Kim D.H., Park S.Y., Park C.H., Kim H.S., Choi S.K., Rew J.S. (2020). Association between Charlson comorbidity index and complications of endoscopic resection of gastric neoplasms in elderly patients. BMC Gastroenterol..

[31] Kinjo K., Matsui T., Hisabe T., Ishihara H., Kojima T., Chuman K., Yasukawa S., Beppu T., Koga A., Ishikawa S. (2018). Risk factors for severity of colonic diverticular hemorrhage. Intest. Res..

[32] Soh H., Chun J., Hong S.W., Park S., Lee Y.B., Lee H.J., Cho E.J., Lee J.H., Yu S.J., Im J.P. (2020). Child-Pugh B or C Cirrhosis Increases the Risk for Bleeding Following Colonoscopic Polypectomy. Gut Liver.

[33] Goto O., Fujishiro M., Kodashima S., Ono S., Niimi K., Yamamichi N., Omata M. (2010). Feasibility of endoscopic submucosal dissection for patients with chronic renal failure on hemodialysis. Dig. Endosc..

